# Improving the Thermostability of *Rhizopus chinensis* Lipase Through Site-Directed Mutagenesis Based on B-Factor Analysis

**DOI:** 10.3389/fmicb.2020.00346

**Published:** 2020-03-03

**Authors:** Zhanbao Jiang, Chengbo Zhang, Minyuan Tang, Bo Xu, Lili Wang, Wen Qian, Jiandong He, Zhihong Zhao, Qian Wu, Yuelin Mu, Junmei Ding, Rui Zhang, Zunxi Huang, Nanyu Han

**Affiliations:** ^1^School of Life Sciences, Yunnan Normal University, Kunming, China; ^2^Engineering Research Center of Sustainable Development and Utilization of Biomass Energy, Ministry of Education, Yunnan Normal University, Kunming, China; ^3^Key Laboratory of Yunnan for Biomass Energy and Biotechnology of Environment, Yunnan Normal University, Kunming, China; ^4^Key Laboratory of Enzyme Engineering, Yunnan Normal University, Kunming, China; ^5^Yunnan Walvax Biotechnology Co., Ltd., Kunming, China

**Keywords:** lipase, thermostability, B-factor analysis, multiple sequence alignment, site-directed mutagenesis

## Abstract

In order to improve the thermostability of lipases derived from *Rhizopus chinensis*, we identified lipase (Lipr27RCL) mutagenesis sites that were associated with enhanced flexibility based upon B-factor analysis and multiple sequence alignment. We found that two mutated isoforms (Lipr27RCL-K64N and Lipr27RCL-K68T) exhibited enhanced thermostability and improved residual activity, with respective thermal activity retention values of 37.88% and 48.20% following a 2 h treatment at 50°C relative to wild type Lipr27RCL. In addition, these Lipr27RCL-K64N and Lipr27RCL-K68T isoforms exhibited 2.4- and 3.0-fold increases in enzymatic half-life following a 90 min incubation at 60°C. Together these results indicate that novel mutant lipases with enhanced thermostability useful for industrial applications can be predicted based upon B-factor analysis and constructed via site-directed mutagenesis.

## Introduction

Lipases (triacylglycerol acyl hydrolases, EC 3. 1. 1. 3) mediate the hydrolysis of triglycerides into monoglycerides, fatty acids, diglycerides, and glycerol at oil-water interfaces (Naik et al., 2010), and can further catalyze interesterification, esterification, alcoholysis, acidolysis, and aminolysis in non-aqueous environments (Pandey et al., 1999; Yu et al., 2014). Owing to their unique catalytic properties, lipases are utilized in a wide range of industrial contexts, such as in the production of food, leather, pharmaceuticals, and bioenergy (Persson et al., 2002; Ferreira-Dias et al., 2013).

Lipases are present in all forms of life, from microbes to mammals. Microbe-derived lipases exist in a wide variety of forms (Gupta et al., 2015). Compared with lipases from animals or plants, microbe-derived lipases can operate across a wider range of temperatures and pH values (Liu et al., 2017). *Rhizopus-*derived lipases have been shown to exhibit superior stability in acidic conditions, making them ideal for industrial applications. Dozens of commercial *Rhizopus* lipases have been produced to date, with *Rhizopus chinensis* CCTCC M201021 being the most commonly used strain for lipase production (Sha et al., 2013). However, lipases derived from *R. chinensis* are most active at moderate temperatures, whereas the reactions catalyzed by lipases in the context of oil processing require higher temperatures (70–90°C) that can lead to the deactivation of lipases lacking heat resistance. As such, it is vital that heat-resistant lipases should be developed so that they may be utilized as superior biocatalysts (Yu et al., 2012a, b).

Many different approaches have been employed to improve lipase kinetic and thermodynamic stability (Bommarius and Paye, 2013; Stepankova et al., 2013; Su et al., 2014). Directed evolution and semi-rational design are the two most common protein engineering approaches used to generate thermostable mutants of target enzymes (Li et al., 2018). Directed evolution through error-prone PCR and DNA shuffling is an effective means of improving the performance of enzymes under high-temperature conditions (Suen et al., 2004; Goomber et al., 2016). Screening through the many colonies necessary to identify superior enzyme isoforms, however, is time-consuming and cannot be effectively performed in host species that grow slowly. An alternative approach to improving enzymatic thermal stability that has been implemented successfully is the B-factor (Xie et al., 2014; Han et al., 2017). This approach relies upon improving thermostability via increasing enzymatic rigidity at certain sites, with B-factors derived from X-ray data offering insight into atom fluctuations and rigidity relative to their equilibrium positions (Ringe and Petsko, 1986). In the present study, we normalized the B-factor values of the Lipr27RCL X-ray structure and thereby identified residues with pronounced flexibility. Following multiple sequence alignment, we then conducted site-directed mutagenesis to improve *R. chinensis* lipase thermostability.

## Materials and Methods

### Materials

*Pichia pastoris* GS115 was from Invitrogen (Shanghai, China). A site-directed mutagenesis kit and DMT chemically competent cells were from TransGen (Beijing, China). The *R*. *chinensis* lipr27RCL gene cloned in the pPIC9K vector was deposited in our laboratory. We obtained 4-nitrophenol palmitate (4-NPP) from Sigma. Mutagenic primers were synthesized by Shuoqing (Kunming, China). All other chemicals were commercially available analytical grade materials.

### Gene Cloning and Site-Directed Mutagenesis

For site-directed mutagenesis experiments, we utilized pPIC9K-*r27RCL* as a template to construct four single-mutant lipase constructs (*Lipr27RCL-K64N, Lipr27RCL-W65I, Lipr27RCL-D66T*, and *Lipr27RCL-K68T*) via introduction of point mutations into *lipr27RCL* through site-directed mutagenesis with a Fast MultiSite Mutagenesis System based on provided directions. The primers for these mutations are compiled in Table 1. We transformed the resultant PCR products into *P*. *pastoris*, and DNA sequencing was used to confirm the identity of the resulting recombinant strains. PCR thermocycler settings were as follows: 5 min at 94°C, then 28 cycles of 30 s at 94°C, 2.5 min at 55°C, and 2 min at 72°C.

**TABLE 1:** Oligonucleotide primers for Lipr27RCL-K64N, Lipr27RCL-W65I, Lipr27RCL-D66T, and Lipr27RCL-K68T.

Primers	Primer sequences
r27RCL-K64N F	5′-GTCGTTCCAGGTACCAATTGGGACTGTAAG-3′
r27RCL-K64N R	5′-GAACATACTTGAGACATTGCTTACAGTCCCAA-3′
r27RCL-W65I F	5′-GTTCCAGGTACCAAGATTGACTGGGACTGT-3′
r27RCL-W65I R	5′-AGGAACATACTTGAGACATTGCTTACAGTCAAT-3′
r27RCL-D66T F	5′-ACCAAGTTGGACTGTACGCAATGTCTCAAG-3′
r27RCL-D66T R	5′-CTTACCATCAGGAACATACTTGAGACATTGCG-3′
r27RCL-K68T F	5′-ACCAAGTGGGACTGTACGCAATGTCTCAAG-3′
r27RCL-K68T R	5′-CTTACCATCAGGAACATACTTGAGACATTGCG-3′

### Protein Expression and Purification

**FIGURE 1:**
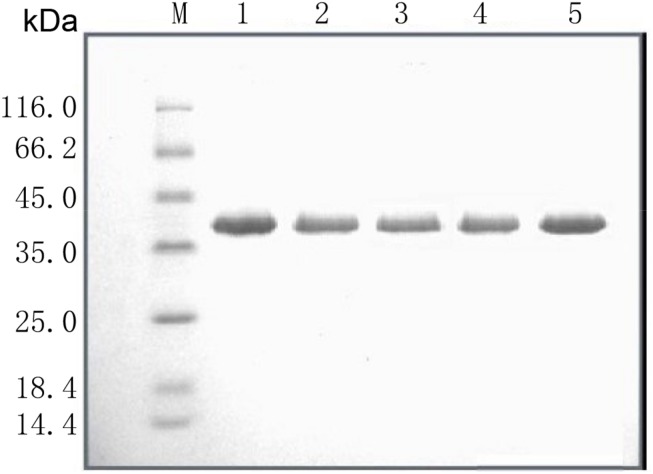
SDS-PAGE analysis of the recombinant lipases. M: standard protein molecular mass markers; Lanes 1–5: Lipr27RCL, Lipr27RCL-K64N, Lipr27RCL-W65, Lipr27RCL-D66T, and Lipr27RCL-K68T, respectively.

*Sal*I was used to linearize the WT pPIC9K/*Lipr27RCL* and mutant constructs, which were then individually transformed into *P. pastoris* GS115 via electroporation. Yeast extract peptone dextrose (YPD) medium supplemented with 200 μg/mL G-418 (Geneticin) was used to select for clones, which were then grown for 2 days at 30°C in a 50 mL volume of buffered glycerol-complex media. This media was then exchanged for 50 mL of buffered methanol-complex media to drive expression of the lipase proteins. Protein purification was conducted using an Amicon centrifugal filter device (cutoff 10.000). Proteins were then sequentially purified via Q Sepharose^TM^ Fast Flow anion exchange column chromatography and Phenyl-Sepharose 4 FF hydrophobic chromatography column chromatography, after which SDS-PAGE was used to confirm purification results (Figure 1 and Supplementary Figure S3), and a protein quantification kit was used to measure enzyme levels.

### Assessment of Enzymatic Activity

We defined one unit of enzymatic activity (1 U) as the quantity of enzyme necessary to mediate the release of 1 μmol p-nitrophenol (p-NP) per minute from 4-NPP (Li et al., 2011). Thermostability was analyzed through assessment of the residual enzymatic activity following incubation at 50°C for 5, 10, 15, 20, 30, 40, 50, 65, 80, 100, and 120 min, and at 60°C for 1, 3, 6, 10, 15, 20, 25, 30, 35, 45, 60, 75, and 90 min at the established optimal pH.

To establish key kinetic parameters for these purified lipases (*K_*m*_, V*_*max*_, and *k*_*cat*_), enzymes were measured in a Tris–HCl (pH 9) buffer solution at 37°C with 4*-*PNN as a substrate provided across a range of concentrations (0.078125–10 mM), with reactions monitored based on p-NP production. Lineweaver–Burk plots were then used to fit the results and to determine these kinetic parameter values (Lineweaver and Burk, 1934).

### Lipase r27RCL Temperature Factor Calculation

B-factor values for all Lipr27RCL Cα atoms were extracted from the PDB file, these B-factors were normalized such that they had a zero mean and unit variance distribution as follows: ${\text{B}}^{{}^{\prime }}=\frac{\text{B}-<\text{B}>}{\sigma \,\left(\text{B}\right)}$, where the ⟨b⟩ is the average of all Cα atoms and σ(B) is the standard deviation of the B-factors for the individual protein (Yuan et al., 2003). We have successfully used these equations in previous studies (Han et al., 2017).

### Circular Dichroism (CD)

Circular dichroism spectra were recorded using a Circular Dichroism (Model: Chirascan, Instrument: 0547) from Applied Photophysics Limited (United Kingdom). All spectra were recorded at 20°C. Conditions, including pathlength: 1 mm, time-per-point: 1 s (25us × 40000), step size: 1 nm, and bandwidth: 1 nm, were used for scanning the 200–250 nm spectra range. CD spectra were collected from the solution of 50 mM sodium phosphate buffer (PBS, pH 7.4) with the lipase concentration of 0.1 mg/ml. After three scans, the final spectrum was corrected by removing the recorded baseline of the PBS control medium.

### MD Simulation Details

The X-ray crystal structure of Lipr27RCL was taken from PDB 6A0W, while structures for the four recombinant lipases Lipr27RCL-K64N, Lipr27RCL-W65I, Lipr27RCL-D66T, and Lipr27RCL-K68T, were constructed using the SWISS-MODEL server (Arnold et al., 2006), using the default parameters. Normal MD simulations of Lipr27RCL, Lipr27RCL-K64N, and Lipr27RCL-K68T were performed at 60°C. After 1000-step energy minimization, all systems were first equilibrated for 5 ns in NVT ensemble and then equilibrated for 5 ns in NPT ensemble by restraining all heavy atoms, and each system was simulated for 30 ns. All systems were solvated with TIP3P waters in an octahedral box, and the minimal distance between each protein and edge of the box was set to 1.0 nm (Jorgensen et al., 1983). Sodium and chloride ions were added with a concentration of 100 nM to neutralize the systems. The GROMACS program suite version 4.5.7 and Amber ff99SB force field were applied in all simulations (Hornak et al., 2006; Hess et al., 2008). All simulations were performed in an isothermal-isobaric ensemble (60°C, 1 bar).

## Results

### Prediction of Mutagenesis Sites Based on B-Factor Analysis

**FIGURE 2:**
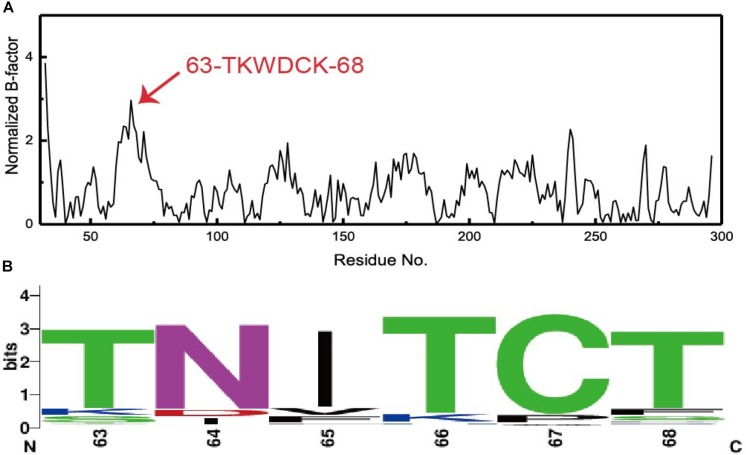
**(A)** Normalized B-factor Lipr27RCL and **(B)** multiple sequence alignment based on 92 lipase sequences from different species of thermophilic fungi.

There is a linear relationship between B-factors determined based upon X-ray diffraction data and the mean square displacement of atoms relative to their average positions. As such, B-factors derived from protein crystal structures offer invaluable insights into the flexibility, stability, and dynamics of individual proteins. In the present study, we extracted and normalized the B-factor values of Cα atoms for Lipr27RCL from its crystal structure (Figure 2A). Residues from T63 to K68 (63-TKWDCK-68) of Lipr27RCL exhibited a high degree of flexibility. Multiple sequence alignment was then performed using 92 lipase sequences derived from thermophilic fungal lipases in the NCBI database, and beneficial sequences from residues 63 to 68 (63-TNITCT-68) were thereby discovered (Figure 2B). Based on the results from this normalized B-factor analysis and multi-sequence alignment, we thus identified K64N, W65I, D66T, and K68T as putative sites that may improve Lipr27RCL thermostability.

### Construction and Characterization of Mutant Lipr27RCL Isoforms

Based on the results from B-factor analysis and multiple sequence alignment, we used site-directed mutagenesis to produce mutant forms of Lipr27RCL in which the 64K, 65W, 66D, and 68K residues had been mutated to 64N, 65I, 66T, and 68T, respectively. The resultant single mutant lipases (K64N, W65I, D66T, and K68T) were termed Lipr27RCL-K64N, Lipr27RCL-W65I, Lipr27RCL- D66T, and Lipr27RCL-K68T for the purposes of this study. These lipases had the same molecular mass (39.50 kDa) as did Lipr27RCL when analyzed via SDS-PAGE (Figure 1). Following purification, we found the specific activities of Lipr27RCL, Lipr27RCL-K64N, Lipr27RCL-W65I, Lipr27RCL-D66T, and Lipr27RCL-K68T to be 2218.52, 2379.71, 1961.14, 2313.66, and 2449.40 U/mg, respectively.

**FIGURE 3:**
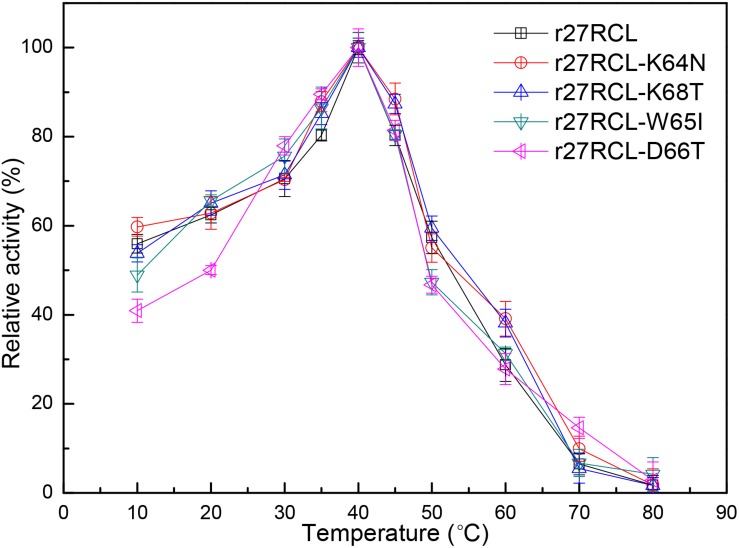
The impact of temperature on recombinant Lipr27RCL, Lipr27RCL-K64N, Lipr27RCL-W65, Lipr27RCL-D66T, and Lipr27RCL-K68T.

**FIGURE 4:**
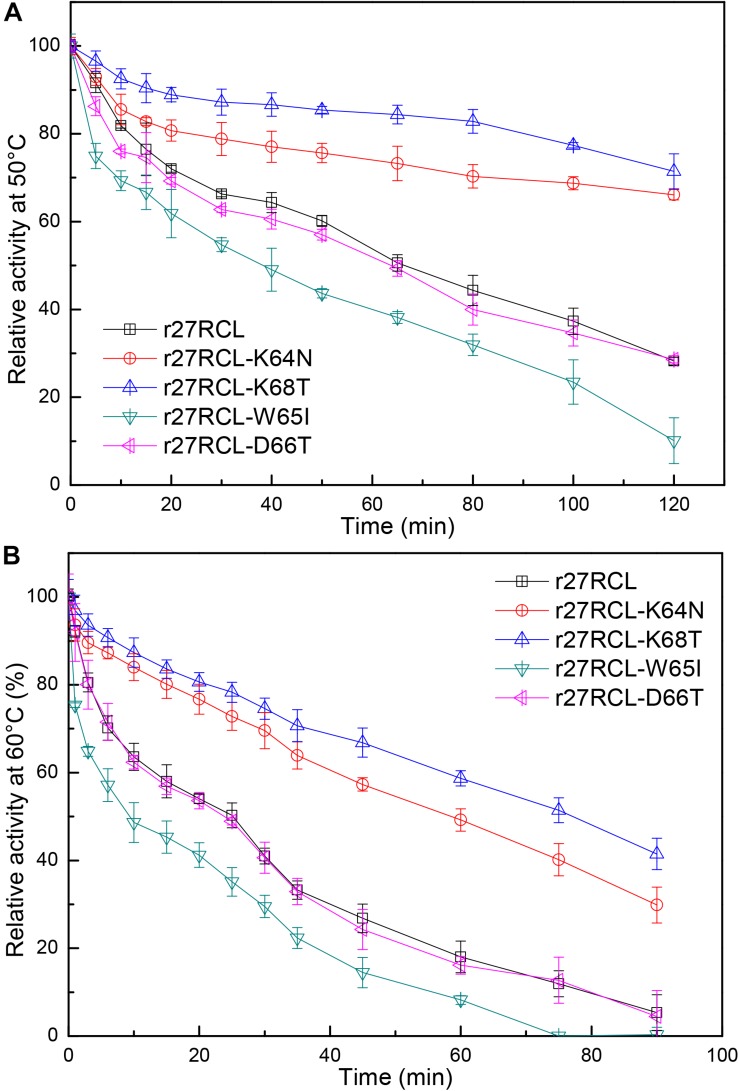
The thermostability of Lipr27RCL, Lipr27RCL-K64N, Lipr27RCL-W65, Lipr27RCL-D66T, and Lipr27RCL-K68T at 50°C **(A)** and 60°C **(B)**.

We next assessed the changes in lipase thermostability as a result of this mutagenic campaign by assessing residual activity of these four Lipr27RCL enzymes and the WT isoform following a 90 min incubation at a range of temperatures. We found that all five of these enzymes functioned best at 40°C (Figure 3). Both Lipr27RCL-K64N and Lipr27RCL-K68T remained stable at 50°C, with residual activities of 66.12 and 76.44% after a 2 h incubation at this temperature, respectively. In contrast, the residual activities of the other lipase isoforms declines substantially under these conditions. Lipr27RCL and Lipr27RCL-D66T had half-lives of 65 min under these conditions, with 28.24 and 28.60% residual activities, respectively, after a 2 h treatment. Lipr27RCL-W65I had a half-life of just 40 min and a residual activity of 10.09% following this 2 h treatment (Figure 4A). At temperatures above 50°C, Lipr27RCL-K64N and Lipr27RCL-K68T were also more stable than the other tested enzymes. Lipr27RCL-K64N retained 49.23% activity following 1 h at 60°C, which was approximately the half-life of this enzyme, and it retained 29.84% activity following a 90 min incubation at this temperature. Lipr27RCL-K68T retained 51.44% activity after a 75 min incubation at 60°C, which was close to its half-life, and it retained 41.47% activity following a 90 min incubation at this temperature. In contrast, Lipr27RCL retained 53.28% activity after only 25 min at 60°C, with just 5.34% activity being retained following 90 min at this temperature. Similarly, Lipr27RCL-D66T retained 48.90% activity after 20 min at 60°C, with 4.44% activity having been retained after 90 min at this temperature. After just 10 min at 60°C, Lipr27RCL-W65I retained just 48.9% activity, with no activity at all remaining after 75 min at this temperature (Figure 4B). Relative to the WT lipase isoform, Lipr27RCL-K64N and Lipr27RCL-K68T exhibited residual activities which were increased by 37.88% and 48.20%, respectively, after 120 min at 50°C, and their respective half-lives had increased by 2.4- and 3.0-fold at 60°C for 90 min. Lipr27RCL-D66T performed nearly identically to WT Lipr27RCL, whereas Lipr27RCL-W65I had poorer thermostability than did WT Lipr27RCL. These findings thus suggested that two of the tested amino acid substitutions (K64N and K68T) were advantageous for Lipr27RCL thermostability.

### Kinetic Analysis of Mutant Lipr27RCL Isoforms

We assessed the kinetic parameters of these five lipase isoforms at a pH of 9.0 at 37°C. The *p*-NP method was used to monitor these reactions, with purified proteins being combined with a range of 4*-*PNN concentrations (0.078125–10 mM). Kinetic measurements revealed that the apparent Michaelis constant (*K*_*m*_) values for Lipr27RCL, Lipr27RCL-K64N, Lipr27RCL-W65I, Lipr27RCL-D66T, and Lipr27RCL-K68T were 0.36, 0.29, 0.45, 0.38, and 0.29 mM, respectively (Table 2). The smaller *K*_*m*_ values for Lipr27RCL-K64N and Lipr27RCL-K68T were indicative of the increased kinetic efficiency of these enzymes relative to WT Lipr27RCL. The catalytic efficiency (*k*_*cat*_/*K*_*m*_) of Lipr27RCL-K64N and Lipr27RCL-K68T was also increased (1.22-fold and 1.24-fold, respectively). The *K*_*m*_ and *k*_*cat*_/*K*_*m*_ values for Lipr27RCL-D66T were similar to those for WT Lipr27RCL. However, the *K*_*m*_ of Lipr27RCL-W65I was 1.25-fold that of Lipr27RCL, with a clear decline in *k*_*cat*_/*K*_*m*_ (Table 2). These kinetic analyses thus revealed that the K64N and K68T substitutions, although primarily intended to improve thermostability, also enhanced the catalytic efficiency and substrate binding of these lipases.

**TABLE 2:** Kinetics of Lipr27RCL, Lipr27RCL-K64N, Lipr27RCL-W65, Lipr27RCL-D66T, and Lipr27RCL-K68T.

Enzymes	*V*_*max*_	*K*_*m*_	*k*_*cat*_	*k_*cat*_/K_*m*_*
	(U/mg)	(mM)	(/s)	(/s/mM)
Lipr27RCL	714.29 ± 23.57	0.36 ± 0.06	188.10 ± 18.21	522.50
Lipr27RCL-K64N	588.24 ± 19.63	0.29 ± 0.09	184.86 ± 18.46	637.45
Lipr27RCL-W65	500.00 ± 23.02	0.45 ± 0.04	131.50 ± 19.38	292.22
Lipr27RCL-D66T	709.00 ± 19.03	0.38 ± 0.05	186.66 ± 16.28	491.21
Lipr27RCL-K68T	714.29 ± 20.21	0.29 ± 0.06	187.95 ± 13.65	648.10

### Circular Dichroism (CD) Spectroscopy

**FIGURE 5:**
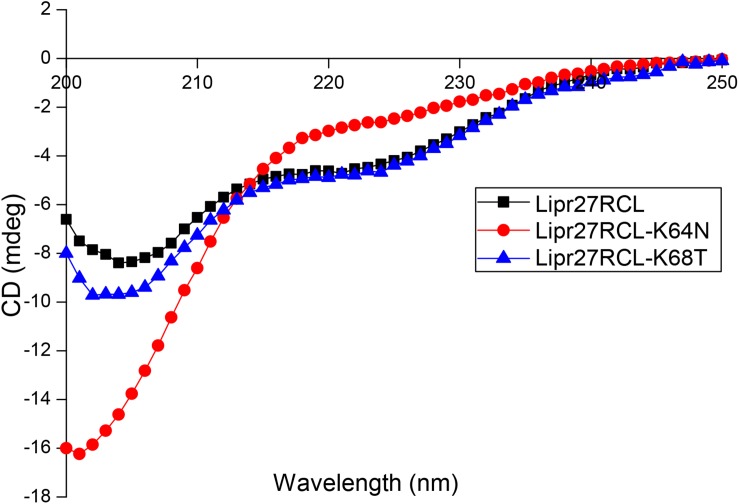
Circular dichroism spectra (CD) of Lipr27RCL, Lipr27RCL-K64N, and Lipr27RCL-K68T.

We selected Lipr27RCL-K64N and Lipr27RCL-K68T with improved thermal stability using CD spectroscopy to compare with Lipr27RCL to prove whether the single mutation caused changes in the secondary structure of Lipr27RCL. CD technology can give information under the protein conformation in solution through the dependence of the optical activity of a peptide chain, with almost no side chain interference (except aromatic amino acids) (Nascimento et al., 2019). The CD spectra of the three lipase isoforms (Lipr27RCL, Lipr27RCL-K64N, and Lipr27RCL-K68T) are shown in Figure 5. In addition, to investigate the thermal denaturation and renaturation of these three isoforms, we heat-treated them at 65°C for 5 min and placed them on ice, and then performed CD spectrum detection. At the same time, the renaturation samples were heat-treated at 65°C and placed on ice, and then recovered at 4°C for 2 days for CD spectrum scan (Supplementary Figure S1).

The CD spectra showed that compared with Lipr27RCL, the ellipticities of the α-helix and β-sheet of the Liper27RCL-K64N isoform changed obviously, while the ellipticity of α-helix of Lipr27RCL-K68T isoform had minor changes with almost no changes of β-sheet (Figure 5). These suggested that mutations at these two single sites might cause changes in the secondary structure of the Lipr27RCL isoform. In addition, the CD spectra of Liper27RCL-K64N and Liper27RCL-K68T were also different, indicating that there were some differences in the thermal stability of the two mutants. Moreover, the CD spectra of the three thermal denaturation isoforms showed that only the ellipticities of β-sheet of the Lipr27RCL-K64N and Lipr27RCL-K68T isoforms changed after heat treatment, while the ellipticities of α-helix and β-sheet of Lipr27RCL both changed (Supplementary Figure S1). These indicated that the thermal stability of Lipr27RCL-K64N and Lipr27RCL-K68T isoforms might be improved. However, the CD spectra of the three isoforms of thermal denaturation and renaturation showed that the ellipticities of the α-helix and β-sheet of these isoforms had little change before and after thermal denaturation and renaturation (Supplementary Figure S1). These indicated that it was difficult for them to recover enzyme activity after denaturation.

### Enhanced Thermal Tolerance of the Single Mutants Explored by MD Simulations

**FIGURE 6:**
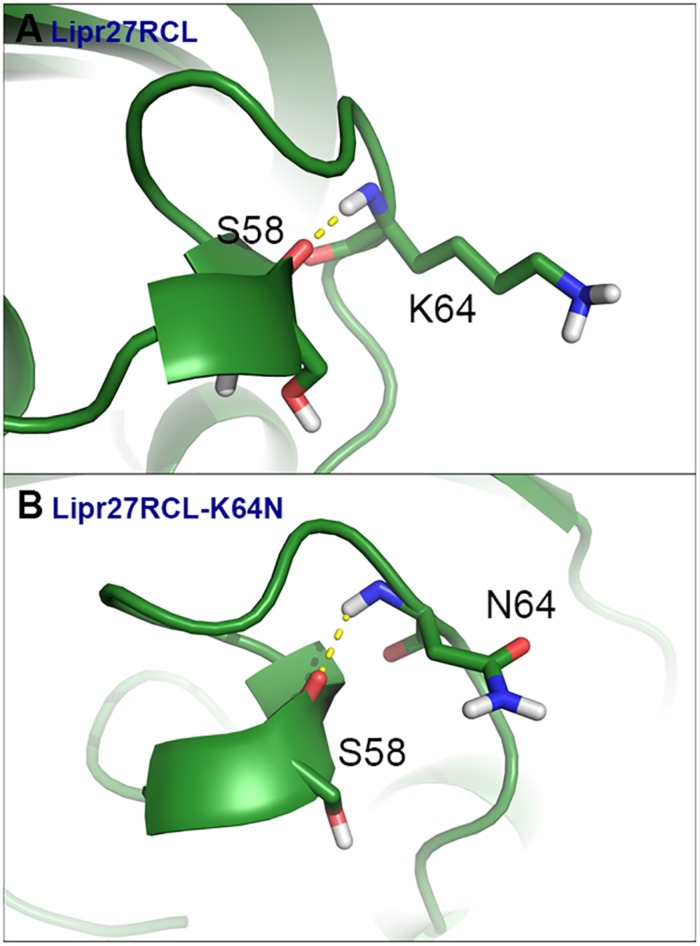
MD simulations Of Lipr27RCL **(A)** and Lipr27RCL-K64N **(B)** conducted at 60°C.

To fully understand the basis of enhanced thermostability in these recombinant lipases, MD simulations for Lipr27RCL and two single mutants (Lipr27RCL-K64N and Lipr27RCL-K68T) with improved thermostability were conducted at 60°C. To investigate the improved thermostability of mutants, novel interactions formed with the mutational sites were monitored during the simulations. It is discovered hydrogen bonding interactions between N64⋯S58 in Lipr27RCL-K64N was stronger than that in K64⋯S58 Lipr27RCL. Specifically, the hydrogen bond forming probability between K/N64⋯S58 were 70.6% and 62.5% in Lipr27RCL-K64N and Lipr27RCL, respectively (Figures 6A,B). Additionally, comparing the root mean square fluctuation (RMSF) values which reflect the flexibility of the mutated residues in the three lipases, we found that RMSF value of the mutated residue N64 in Lipr27RCL-K64N was smaller than that of K64 in Lipr27RCL, indicating improved stability of the mutated residue (Table 3). The side chain of N64 is smaller than that of K64, and as a result N64 exhibited increased local stability owing to its mild floating amplitude, and this coupled with the retention of normal hydrogen bond interactions led to the enhanced thermostability of Lipr27RCL-K64N.

**TABLE 3:** RMSF for mutated residues over the whole simulation in Lipr27RCL and two mutants.

Enzymes	K/N64 (nm)	K/T68 (nm)
Lipr27RCL	0.1576	0.1995
Lipr27RCL-K64N	0.1187	0.1786
Lipr27RCL-K68T	0.1636	0.1478

Similarly, RMSF value of the mutated residue T68 in Lipr27RCL-K68T was smaller than that of K68 in Lipr27RCL, and the smaller RMSF value of T68 indicate the mutated site was relative stable during simulation (Table 3). T68 in Lipr27RCL-K68T also had a shorter side chain relative to that of K68, and the α-helix where T68 located was thus more stable than that of the wild type enzyme.

## Discussion

The development of thermostable lipases has clear and practical potential to meet with industrial demands. Furthermore, generating such stable lipases may simultaneously improve their kinetic efficiency (Rogers and Bommarius, 2010; Han et al., 2017). In the present study, we utilized a B-factor comparison and multi-sequence alignment strategy to guide our efforts to design and produce thermostable lipase mutants. The resultant Lipr27RCL-K64N and Lipr27RCL-K68T proteins, which were designed via site-directed mutagenesis, exhibited both higher thermostability and superior catalytic efficiency.

Normalized B-factor analysis of Lipr27RCL was the key step in the design of thermostable recombinant isoforms of this enzyme, given that B-factors offer insight into protein fluctuations, which are indicative of atom rigidity relative to their corresponding positions (Ringe and Petsko, 1986), and previous work has shown that improved thermostability depends upon achieving a higher degree of rigidity (Podar and Reysenbach, 2006). As such, we analyzed normalized B-factor values from Lipr27RCL, and aligned this sequence with those of other thermophilic lipases using sequences from NCBI. We were thereby able to identify residues with pronounced degrees of flexibility, and we then constructed recombinant mutant lipases (Lipr27RCL-K64N, Lipr27RCL-W65I, Lipr27RCL-D66T, and Lipr27RCL-K68T) in which these flexible residues had been mutated. In subsequent thermostability and kinetic analyses, we found that Lipr27RCL-K64N and Lipr27RCL-K68T exhibited superior thermostability, substrate affinity, and catalytic efficiency. As such, these results confirm that B-factor analysis can facilitate efforts to selectively improve lipase thermostability profiles.

Based on sequence and structural analyses, we found further evidence suggesting that Lipr27RCL-K64N and Lipr27RCL-K68T may exhibit greater thermostability relative to other untested lipase isoforms, leading us to focus our in depth characterization efforts on these isoforms. We had also constructed a quadruple mutant lipase (K64N, W65I, D66T, and K68T) for this study, but found its thermostability to be 40% lower than the WT Lipr27RCL in a matter of minutes. This is likely at least in part due to the loss of hydrogen bond network interactions introduced by the I65 mutation, thereby adversely impacting the formation of hydrogen bond networks and the α-helix around N64 and T68. This loss of hydrogen bond networks in turn reduced the regional thermostability, markedly decreasing the thermostability of this quadruple mutant lipase. As such, other lipase isoforms containing the I65 mutation will similarly not display improved thermostability owing to this loss of hydrogen bond network interactions. Similarly, lipase isoforms bearing T66 mutations are likely to exhibit limited thermostability due to the lack of stable interactions between this residue and other surrounding residues. As such, the characterization of lipase isoforms containing mutated I65 or T66 residues was not tested in this work. However, the double mutant lipase (Lipr27RCL-K64N/K68T) exhibited cumulative thermostability of Lipr27RCL-K64N and Lipr27RCL-K68T (Supplementary Figure S2), indicating that the role of the two mutation sites may be aggregate.

In summary, we found that we were able to produce novel mutant lipases (Lipr27RCL-K64N and Lipr27RCL-K68T) with improved thermostability through a combination of B factor analysis and site-directed mutagenesis, and these resultant enzymes represent attractive candidates for use in industrial applications.

## Data Availability Statement

The raw data supporting the conclusions of this article will be made available by the authors, without undue reservation, to any qualified researcher.

## Author Contributions

CZ performed the major experiments containing enzyme production, purification, and CD analysis. ZJ and MT carried out the experiments containing site-directed mutagenesis and characterization of lipases. LW, WQ, JH, and ZH helped with protein purification. NH analyzed the B-factor and MD simulation. QW and YM prepared experimental materials. BX, JD, and RZ dealt with the problems encountered in the experiment and coordinated the study. CZ wrote the manuscript. ZH and NH revised this manuscript. All authors read and approved the final manuscript.

## Conflict of Interest

LW, WQ, JH, and ZZ were employed by Yunnan Walvax Biotechnology Co., Ltd. The remaining authors declare that the research was conducted in the absence of any commercial or financial relationships that could be construed as a potential conflict of interest.

## Supplementary Material

FIGURE S1 Circular dichroism spectra (CD) of Lipr27RCL **(A)**, Lipr27RCL-K64N **(B)**, and Lipr27RCL-K68T **(C)** under the condition of thermal denaturation at 65°C for 5 min and renaturation at 4°C for 2d, respectively.

FIGURE S2 The thermostability of Lipr27RCL, Lipr27RCL-K64N, Lipr27RCL-K68T, and Lipr27RCL-K64N/K68T at 60°C.

FIGURE S3 The original image of Figure 1.
