# Supplementary figures and images for: Improving the Thermostability of Rhizopus chinensis Lipase Through Site-Directed Mutagenesis Based on B-Factor Analysis
Source: Front Microbiol. 2020 Mar 3;11:346. doi: 10.3389/fmicb.2020.00346 (PMC7063977; doi:10.3389/fmicb.2020.00346)

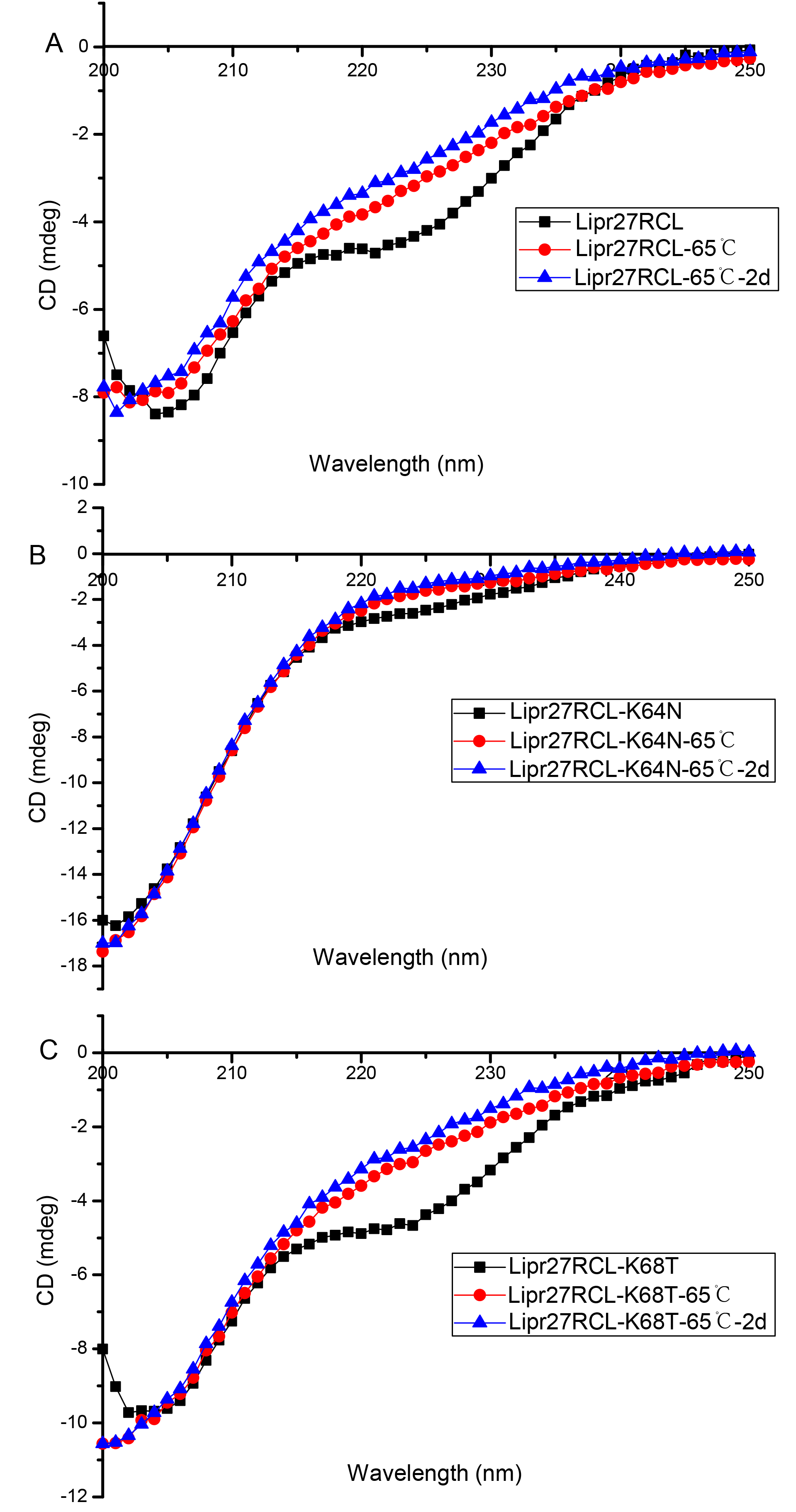

Supplement: FIGURE S1 — Circular dichroism spectra (CD) of Lipr27RCL (A), Lipr27RCL-K64N (B), and Lipr27RCL-K68T (C) under the condition of thermal denaturation at 65°C for 5 min and renaturation at 4°C for 2d, respectively. [file Image_1.TIF]

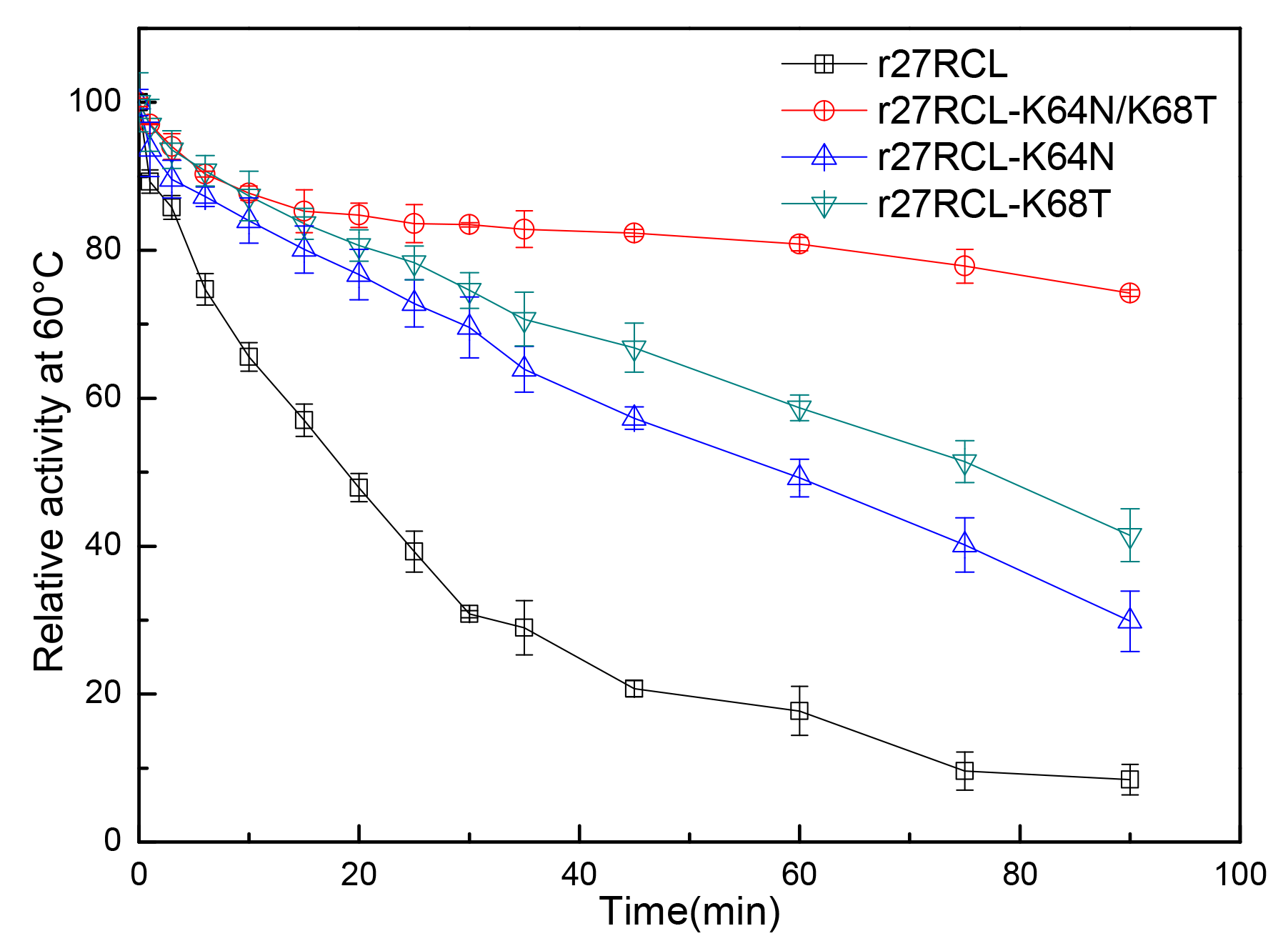

Supplement: FIGURE S2 — The thermostability of Lipr27RCL, Lipr27RCL-K64N, Lipr27RCL-K68T, and Lipr27RCL-K64N/K68T at 60°C. [file Image_2.TIF]

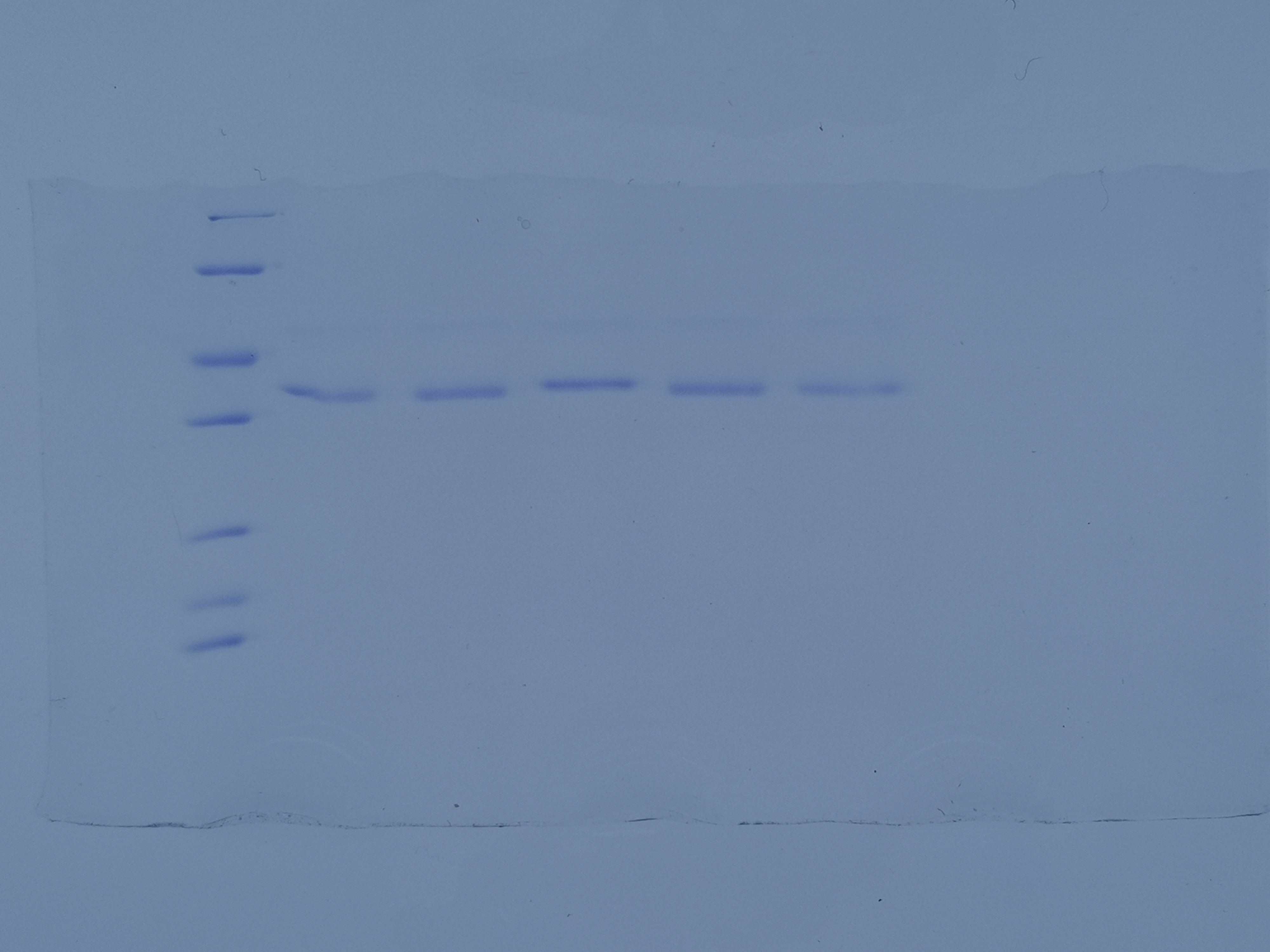

Supplement: FIGURE S3 — The original image of Figure 1. [file Image_3.TIF]
